# Comparative Genomics and Gene Pool Analysis Reveal the Decrease of Genome Diversity and Gene Number in Rice Blast Fungi by Stable Adaption with Rice

**DOI:** 10.3390/jof8010005

**Published:** 2021-12-22

**Authors:** Qi Wu, Yi Wang, Li-Na Liu, Kai Shi, Cheng-Yun Li

**Affiliations:** 1 State Key Laboratory for Conservation and Utilization of Bio-Resources in Yunnan, Yunnan Agricultural University, Kunming 650201, China; wuqi_ynkm@ynau.edu.cn (Q.W.); wyi_0114@ynau.edu.cn (Y.W.); handyliu@126.com (L.-N.L.); 2 College of Science, Yunnan Agricultural University, Kunming 650201, China; 3 Yunnan Organic Tea Industry Intelligent Engineering Research Center, Key Laboratory of Intelligent Organic Tea Garden Construction in Universities of Yunnan Province, Key Laboratory for Crop Production and Smart Agriculture of Yunnan Province, Yunnan Agricultural University, Kunming 650201, China; 4 Key Laboratory of Green Prevention and Control of Agricultural Transboundary Pests of Yunnan Province, Agricultural Environment and Resources Institute, Yunnan Academy of Agricultural Sciences, Kunming 650201, China; 5 School of Foreign Language, Yunnan Agricultural University, Kunming 650201, China; sksdasda@163.com

**Keywords:** *Magnaporthe oryzae*, comparative genomics, gene pool, selective sweep, host adaption

## Abstract

*Magnaporthe oryzae* caused huge losses in rice and wheat production worldwide. Comparing to long-term co-evolution history with rice, wheat-infecting isolates were new-emerging. To reveal the genetic differences between rice and wheat blast on global genomic scale, 109 whole-genome sequences of *M. oryzae* from rice, wheat, and other hosts were reanalyzed in this study. We found that the rice lineage had gone through stronger selective sweep and fewer conserved genes than those of *Triticum* and *Lolium* lineages, which indicated that rice blast fungi adapted to rice by gene loss and rapid evolution of specific loci. Furthermore, 228 genes associated with host adaptation of *M. oryzae* were found by presence/absence variation (PAV) analyses. The functional annotation of these genes found that the fine turning of genes gain/loss involved with transport and transcription factor, thiol metabolism, and nucleotide metabolism respectively are major mechanisms for rice adaption. This result implies that genetic base of specific host plant may lead to gene gain/loss variation of pathogens, so as to enhance their adaptability to host. Further characterization of these specific loci and their roles in adaption and evaluation of the fungi may eventually lead to understanding of interaction mechanism and develop new strategies of the disease management.

## 1. Introduction

*M. oryzae* is an ascomycete pathogen that causes blast disease in more than 50 species of plants, especially many economic cereal crops such as rice, wheat, barley, millet, and oats [1,2,3,4,5,6]. Furthermore, some grasses including goosegrass, annual ryegrass, perennial ryegrass, tall fescue, and St. Augustine grass can also become hosts for it [7]. In the past 400 years, people used to focus on the rice blast disease management because of its great threat to rice, which is an important crop to human beings. However, unlike the rice blast, the epidemic of wheat blast disease caused by *M. oryzae* was first recorded in Brazil in 1985 followed by its spread to the United States in 2011 and then to Bangladesh [8,9,10,11,12]. Blast fungi threaten productions of two main crops, which indicates its significant economics, thus it is urgent to explore the genetic difference among *M. oryzae* populations. Recently, research showed that the population structure of *M. oryzae* is subdivided into multiple lineages with limited host range [13]. Southeast Asia is the center of origin, diversity, and dispersion of the rice blast fungus. Except for the complex population structure of epidemic isolates in Southeast Asia, most of the epidemic isolates in Europe and Africa come from the same lineage [14]. The adaptive evolution of wheat-infecting isolates to host is complicated, some of which have clustered with *Lolium*-infecting isolates. At the same time, although rice-infecting isolates around the world are subdivided into different lineages, their diversity was lower than that of wheat-infecting isolates [13,15], which indicates that they are well-adapted to rice and have relatively stable genome.

During the co-evolution between plant and pathogen, the evolution of pathogens was directly related to the differentiation of hosts [16]. Due to domestication of crops with morphological and genetic variation, pathogens are subject to selection pressures from their hosts. Strong positive (natural) selection of a beneficial allele could lead to the selective sweep of adjacent linked sites, and eventually decreases of genetic diversity [17]. It is generally believed that positive selection plays an important role in the outcome of new phenotypes, and balancing selection is regarded as the main mechanism to maintain population diversity [18,19,20]. Compared with the core genome, many effector genes in pathogens present a trend of accelerated evolution to gain advantages in the arms race with their hosts. The RxLR effectors in *Phytophthora sojae* and Hopz family of type III secreted effectors in *Pseudomonas syringae* showed high polymorphism and positive selection during co-evolution with host [21,22]. Previous studies have showed that the selective sweep can have a significant impact on the level of population subdivision, especially when the sweep has not yet spread to all populations within one species [23,24,25,26]. For example, the genes in the strongest sweep region involving biotic and abiotic responses were found in the formation of two clusters of *Rhynchosporium commune* from Central Europe and Ethiopia, respectively [27].

Population genetic analysis of 446 wild rice and 1083 indica and japonica genomes showed that selective sweeps were accompanied with the whole domestication process and population subdivision in rice [28]. Rice and *Setaria* infecting isolates were diverged followed by rice domestication [13]. Therefore, there is a long co-adaption history between *M. oryzae* and rice, and whether the events of selective sweeps happened in population of rice-infecting isolates needs to be discovered. In this study, population genetic statistics calculated on 10 kb window showed that compared with the alternating distribution of two different characteristic regions in the genome of wheat-infecting isolates, rice-infecting isolates were under selective sweep at the whole genome level and showed lower diversity. In addition, gene pool analysis indicated that rice-infecting isolates contained fewer genes than wheat- and *Lolium*-infecting isolates, and discovered some candidate loci of *M. oryzae* adapted to host and potential virulence factors at the same time. Our results showed that this comparison could be helpful to understand genomic instability, putative target genes for host adaptation and to understand how stable adaption with host was realized to achieve a balanced population.

## 2. Materials and Methods

### 2.1. Genome Sequences

One hundred and nine published genome data of *M. oryzae* from eleven host genera were downloaded from the National Centre of Biotechnology Information assembly database (http://www.ncbi.nlm.nih.gov/assembly, accessed on 10 December 2021, Table S1) [10,11,12,13,16,29,30,31,32,33,34,35,36,37,38,39,40]. Among them, there were 63 rice-infecting isolates (the FR13, GUY11 and FJ81278 had both NGS and SMRT assembly data) and 43 non-rice-infecting isolates. In the population genetic statistics and gene pool analysis between different lineages of *M. oryzae*, 48 isolates were used. Among them, 24 non-rice-infecting isolates, including 13, 6, 2, 2, and 1 isolates from *Triticum aestivum*, *Lolium perenne*, *Lolium multiorum*, *Bromus tectorum,* and *Triticum durum* respectively, were combined into non-rice lineage (NRL) and compared with rice lineage (RL) which was composed of 24 rice-infecting isolates (Table S1, Figure S1). As a supplement, seven isolates of *Eleusine* lineage (EL) were used to verify the conclusions obtained from population genetic analysis. In order to reveal the PAV of genes in rice and non-rice isolates, the 24 isolates of RL were selected according to the rank of genome size from NCBI database with a total ungapped length of each genome sequence over 39.0 Mb. These rice-infecting isolates came from 8 countries in South America, North America, Europe, Asia, and Africa, ranging from 1988 to 2015, while wheat-, *Lolium*-, and *Bromus*-infecting isolates come from Brazil, Bolivia, Paraguay, the United States and Bangladesh, ranging from 1988 to 2016. In the Figures S2, S4, S6 and S8, it could be seen that when comparing RL with different non-rice lineages of different population sizes, the selected isolates were distributed in the major branches of the phylogenetic tree constructed by SNP dataset of 148 global distributing rice-infecting isolates (all the genomes submitted to the NCBI assembly database before May 2020) rather than coming from a single branch. Other isolates in the Table S1 were used together with the 48 isolates mentioned above to analyze the diversified distribution patterns of 11 cloned *AVR* genes in *M. oryzae* from different hosts, and served as assistants and contrasts in gene pool analysis. Two *Stenotaphrum*-infecting isolates were included as outgroups. The reference genome 70-15 version 8 and other two Magnaporthaceae species (*Gaeumannomyces graminis* var. *tritici* and *Magnaporthe poae*) were downloaded from the Broad Institute (https://www.broadinstitute.org/scientific-community/science/projects/fungal-genome-initiative/magnaporthe-comparative-genomics-proj, accessed on 10 December 2021).

### 2.2. Single-Nucleotide Polymorphism Discovery and Population Genetics

The genome-to-genome SNP calling approach [34] was used in this study as the pair-end reads for 27 published genome sequences of the 48 isolates were not obtained online. First, each assembly sequence was mapped to the reference genome by a program NUCmer of MUMmer v3.23 with parameters -maxmatch –c 100 [41]. Final SNP data were obtained by program show-snps with parameter -Clr. Our results showed that a total of 6.5 M SNPs obtained from the 48 genomes compared with 70-15, which covered 547.9 K sites of reference genome, were identified for population structures (Table S2). Due to the close genetic relationship between BdJes16-1, BdMeh16-1, BdBar16-1, and B71, we also carried out the genome-to-genome SNP calling again with the sequences spliced to chromosome level of B71 (GCA_004785725.2) as reference genome. It could be seen that except BdJes16-1, BdMeh16-1 and BdBar16-1, which only contain 679, 981, and 1212 SNPs, other isolates of NRL contain much more SNPs (ranging from 52.6–127.0 K, average 104.1 K, covering 350.9 K sites of B71) than those of rice-infecting isolates again with 70-15 (ranging from 10.3–23.6 K, average 13.0 K, covering 77.2 K sites of 70-15). These results indicate that there are high differences among these isolates of NRL, which can support our comparative genomic analysis later. Based on the SNP dataset, VariScan v2.0.3 was used to calculate nucleotide diversity (π) and Tajima’s *D* [42] with PHYLIP input data files and parameter RunMode = 12, and population differentiation (*F*_st_) was calculated by Genepop v4.7.2 [43] with MenuOptions = 6.2. All data were estimated with the 10 kb windows according to the reference genome. The grouping-specific SNPs (S-SNPs) which were all X in RL and Y in NRL (X, Y is one of A, T, G, and C) were screened out based on the occurrence frequency of SNPs at the same site in RL and NRL. The dS values were calculated by Paml v4.9c with yn00 model [44].

### 2.3. Gene Prediction, Annotation, and Secreted Proteins Prediction

Protein-coding genes were predicted by Augustus V3.0.3 [45]. The *Magnaporthe grisea* model was selected for Augustus prediction. Predicted genes with deduced protein length shorter than 50 amino acids, or overlapping with repeat sequences for more than 50% of their transcript length were removed. Some genes apparently belonging to the rice genome had also been eliminated. Protein sequences were functionally annotated against the EggNOG database [46]. The putative secreted proteins were identified by SignalP 3.0 [47]. The PAV of *AVR* genes were detected by searching the nucleotide sequence against each isolate with BLASTN (*E* < 1 × 10^−10^) [34].

### 2.4. Gene Pool Analysis

Homologous genes with sequence identities of 100%, 80–100%, and 50–80% were defined as identical, similar, and divergent, respectively, while those below 50% were considered non-homologous [48]. We performed BLASTP (*E* < 1 × 10^−5^) comparison on the protein-encoded genes predicted by Augustus in each genome, and removed the homologous genes with similarity more than 50%. The gene pool of 48 *Magnaporthe* isolates with 11,914 genes was obtained. The PAV of genes in the gene pool were detected by searching the protein sequences against each isolate with TBLASTN (*E* < 1 × 10^−10^), and the genome would contain the gene when the sequence identity and matching overlap were above 50% [16]. To identify genes of *Magnaporthe* under selection from different hosts, their present frequencies in different lineages were derived. The significance of the differences of the presence frequencies for each gene between the two lineages was determined by the Fisher’s exact test. The resulting raw *p* values of 1599 identified PAVs were then corrected via false discovery rate (FDR). As the number of isolates we used for population comparison was small, genes with significantly different frequencies (FDR < 0.0001) were identified as those related to host adaptability of *M. oryzae* to lock the candidate gene as accurately as possible. The maximum-likelihood trees were constructed based on the SNP data with default parameter values by FastTree v2.1.9 [49] or the binary PAV data with 1000 bootstraps by IQ-TREE v2.1.4 [50], and displayed with FigTree v1.4.3 (http://tree.bio.ed.ac.uk/software/figtree/, accessed on 15 December 2021).

### 2.5. Indentification of Expressed Genes

We used RNA-Seq data from samples 2, 5, 7, and 12 from Bangladesh wheat fields to identify the expression of grouping-specific genes of non-rice lineage, including comparisons between asymptomatic and symptomatic samples [12]. The specific genes expressed only after the symptoms were considered to be related to the host adaptability of wheat isolates. In addition, the RNA-Seq data of 98-06′s mycelium and conidia infection at 0, 8, 24, 48, and 72 h post inoculation were used to compare the expression of grouping-specific genes of rice lineage *in planta* and in vitro culture [31]. The genes were selected according to two situations, the first is that genes were not expressed in one situation of *planta* or culture, the second is that genes showed differential expression.

### 2.6. Statistical Analysis

The statistical tests used are illustrated in the article and the figure legends. To be more precise, since the data did not conform to the normal distribution, we performed Spearman correlation analysis between the number of genes contained in the isolate and the four genomic features (total length, total ungapped length, number of sequences and N50). At the same time, Mann–Whitney U test was used to analyze whether there were significant differences in the four genomic features mentioned above between rice- and non-rice-infecting isolates. Fisher’s exact test with FDR corrected for multiple comparisons was used in the search for specific genes of different lineages.

### 2.7. Data Availability

The data to confirm the conclusions are completely listed in the article and Supplementary Materials. The NCBI database accession number of the genome used for analysis is showed in Table S1. Detailed information such as predicted protein sequences, occurrence frequencies in different lineages, and gene function annotations of the 228 genes associated with host adaptation of *M. oryzae* are recorded in Table S4.

## 3. Results

### 3.1. Population Genomic Analysis of Rice- and Non-Rice-Infecting Isolates

Previous studies showed that the wheat-infecting isolates have closer genetic relationship with *Lolium*-and *Brome*- than that of rice-infecting isolates. Wheat-infecting isolates were divided into two lineages when phylogenetic trees were built by analyses of single copy gene and pairwise distance. The *Triticum* lineage (TL) consists almost all of wheat-infecting isolates and the *Lolium* lineage (LL) comprises *Lolium*-, *Festuca*-, and *Brome*-infecting isolates besides several wheat-infecting isolates [13]. Compared with other host infecting isolates, rice-infecting isolates seem to possess strict host specificity and showed lower diversity [13,51]. To better understand the selection effect of rice on *M. oryzae*, the genetic divergence between 48 rice and non-rice isolates were analyzed. When the phylogenetic tree was constructed with genome-wide SNP data, it could be seen that isolate from same host were in the same branch except a few wheat-infecting isolates which was consistent with previous study (Figure S1).

**Figure 1:**
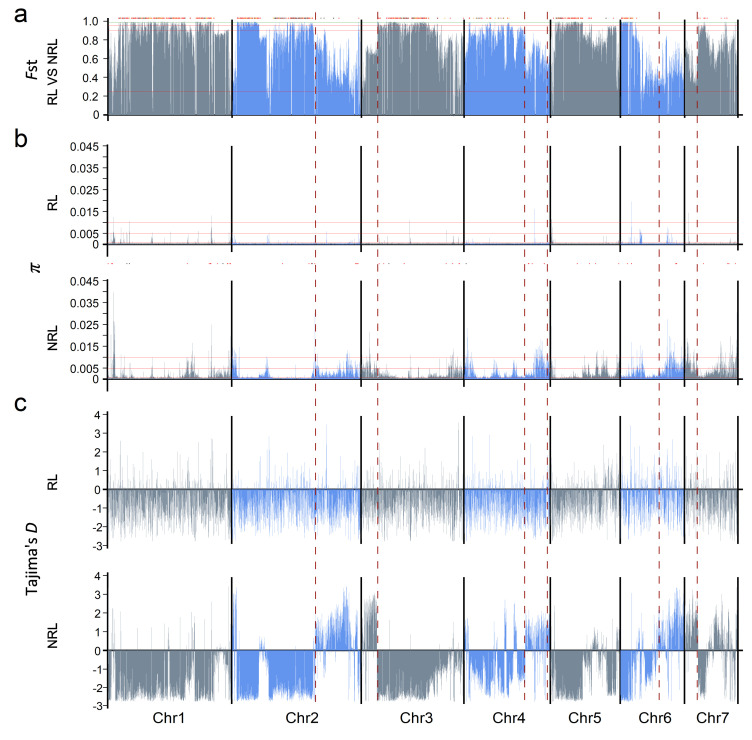
Genome–wide view in 10 kb windows of differentiation (*F*_st_), diversity (*π*) and Tajima’s *D*. (**a**) The *F*_st_ compared between RL and NRL. The RL included 24 rice-infecting isolates with the largest ungapped length of genome in NCBI database, and the NRL included 14 wheat-, 8 *Lolium* and 2 *Bromus*-infecting isolates. The locations of 856 genes mutated by S-SNPs were showed at the top of *F*_st_. The orange dot indicated that there was at least one S-SNP is in CDS, the red dot indicated that non-synonymous substitutions had occurred in CDS, and the black dot indicated that all S-SNPs were located in intron or untranslated region. The distributions of *π* (**b**) and Tajima’s *D* (**c**) of RL and NRL. The area sandwiched by the red dotted line in the vertical direction represented the area enriched by window of Tajima’s *D* < 0 in NRL. In these regions, the *π* of NRL increased while the *Fst* between RL and NRL decreased. The dots between *π* of RL and NRL in (**b**) were the favorable genes present in 70-15. Among them, red and black indicated favorable genes of RL and NRL respectively.

To reveal the genetic diversity and differentiation between RL and NRL, *F*_st_, *π,* and Tajima’s *D* were used. The average *F*_st_ value of all 4045 windows was 0.73, indicating that there was high genetic differentiation between the two groups (Figure 1a). Moreover, the *π* and Tajima’s *D* also showed different patterns between the two groups, indicating that RL had lower diversity than that of NRL (Figure 1b,c). When considering *π* > 0.001, more windows were found in NRL (58.9%) than that in RL (5%, Figure S2a–c). When calculating the diversity of intragene and intergenic regions, the diversity of RL was also the lowest among all groups or lineages, which was similar to the previous results (Table 1) [13]. In addition, the windows with Tajima’s *D* > 0 were estimated from RL (81.3%) and NRL (71.8%). The distribution patterns of windows with Tajima’s *D* > 0 or <0 were obviously different between the two groups (Figure 1c and Figure S2d–f). For NRL, more enriched windows with Tajima’s *D* > 0 or <0 seemed to cluster in certain regions on 2–7 chromosomes. Compared with the regions enriched by windows with Tajima’s *D* < 0, most of the regions enriched by windows with Tajima’s *D* > 0 showed higher diversity and lower *F*_st_ at the same time, which indicated that balancing selection played a dominant role in these regions, and there were many alleles with medium frequency. The *F*_st_ of most windows in these regions was still higher than 0.25, suggesting that there was a great genetic differentiation between RL and NRL [52]. When using NRL after removing BdJes16-1, BdMeh16-1, and BdBar16-1 and RL of the same population size to calculate the *F*_st_, π, and Tajima’s *D*, the results were similar to those before removing (Figures S3 and S4). Other similar results were also obtained when we compared RL with TL and LL, respectively (Figure S5–S8). In addition, after comparing the *F*_st_ between different lineages, it was found that the degree of genetic differentiation between rice linage and non-rice lineages was significantly higher than that between non-rice lineages (Figure S9a,b). At the same time, the diversity of RL was significantly lower than that of non-rice lineages with the same number of isolates, and EL had the highest diversity among all the non-rice lineages (Figure S9c). These results indicated that some regions in the genomes of NRL were influenced by the balance selection and showed significantly higher diversity than RL. However, the lower diversity in RL implied that in the long history, breeding of rice varieties has brought strong selection, which might lead the genetics of rice infecting isolate populations toward simplification and specificity.

### 3.2. SNPs with Host Selection

**Figure 2:**
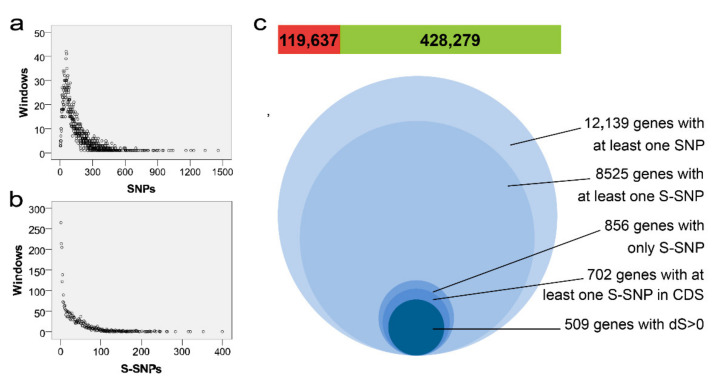
Windows and Genes affected by SNPs. Distribution of windows on chromosome against the corresponding SNPs (**a**) and S-SNPs (**b**). (**c**) The number of sites in the two classes and the genes affected by different mutations. The numbers in the green and red bars indicated the number of identified sites for S-SNPs and SNPs, respectively. Circles with different sizes represented the number of genes with different mutations due to SNP.

According to previous genetic differentiation analysis of *M. oryzae* population, 1411 windows were found based on *F*_st_ > 0.9 and 97.7% of windows contained 1–1500 SNPs (Figure 2a). A total of 119,637 S-SNPs covering 8525 genes were obtained and 510 windows (12.6%) did not contain S-SNPs (Figure 2b,c). Among 8525 genes possessing at least one S-SNP, 856 genes only contained S-SNPs, and 7669 genes contained both S-SNPs and moderate-frequency alleles. There were 702 genes with S-SNPs distributed in coding sequences (CDS), and 509 of which with dS > 0. Among these 856 genes with S-SNPs, it could be seen that almost all the genes (84.2%) were distributed in the windows with *F*_st_ > 0.9, a few genes (15.8%) were found in several regions of all chromosomes with *F*_st_ < 0.9 (Figure 1a), especially the last third of chromosome 2. Therefore, a large number of S-SNPs involved in genetic differentiation between RL and NRL were widely distributed in the genomes, indicating that these two groups were evolving toward separate trajectories, and it was difficult to find out the genes related to the adaptability of *M. oryzae* by population genetic statistics or association analysis based on the SNP dataset.

### 3.3. Highly Polymorphic Distribution of 11 Cloned AVR Genes in Two Groups

**Figure 3:**
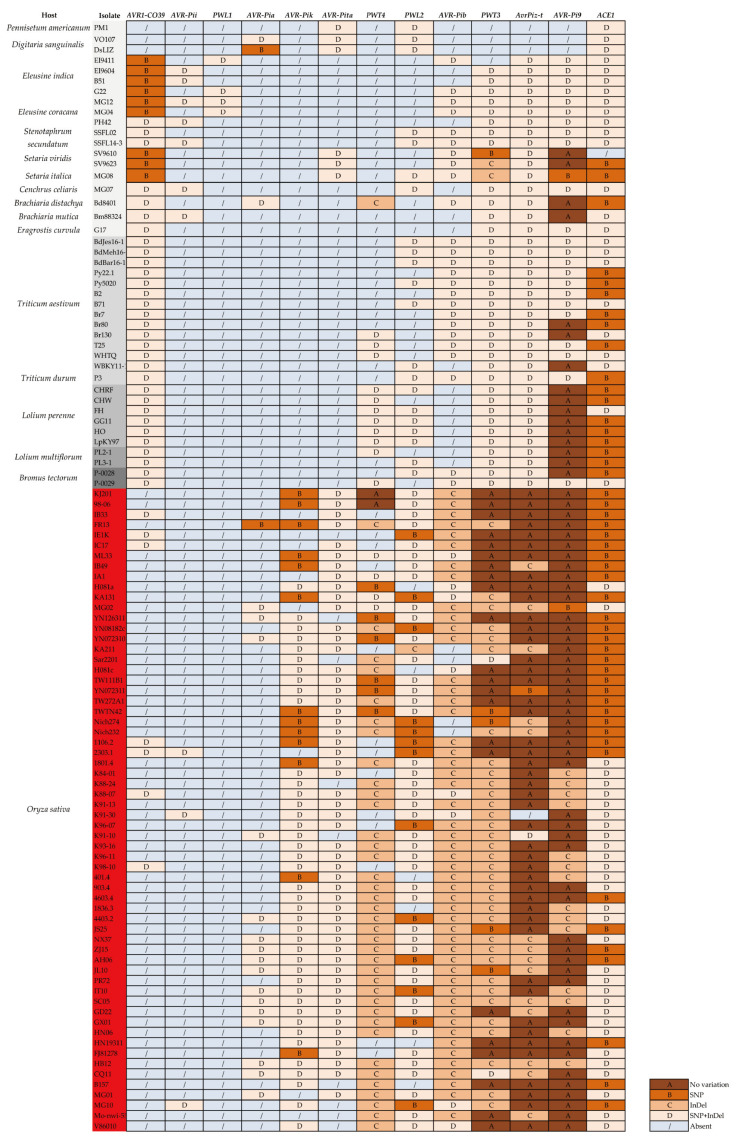
Highly polymorphic distribution of 11 cloned *AVR* genes in *M. oryzae* isolates from different hosts. The isolates with red color came from rice, and the gray from light to dark indicated wheat-, *Lolium*- and *Bromus*-infecting isolates. The other non-rice-infecting isolates were not filled with color.

Rice and *M. oryzae* were regarded as an ideal model for studying host-pathogen interaction. The arms race between *R* and *AVR* genes were involved in the recognition between host and pathogen. There were more than 40 *AVR* genes identified in *M. oryzae*. Eleven *AVR* genes have been cloned and characterized by their cognate Rgenes, including *PWL1* [53], *PWL2* [54], *AVR-Pita* [55], *ACE1* [56], *AVR-Pia*, *AVR-Pii*, *AVR-Pik* [57], *AvrPiz-t* [58], *AVR1-CO39* [59], *AVRPib* [60], and *AVRPi9* [61]. Except for 48 genomes used for SNP analysis, 57 *Magnaporthe* genomes from 11 different hosts were added to select the *AVR* genes that were involved in the genetic differentiation together (Figure 3).

When an isolate contains the detected *AVR* gene, we would distinguish and label the variant types of the gene with the cloned *AVR* genes. No variation, SNPs, InDel, and the combination of SNPs and InDel were marked as A, B, C, and D respectively. When describing the genotypes of *PWT3* or *PWT4* in different strains, A indicated that the sequences in this strain were consistent with those in 70-15 or Ina168 respectively. According to the sequence alignment results, the distribution patterns of the 11 *AVR* genes in RL and NRL could be divided into four types. First, as this gene was widely distributed in NRL and rarely appeared in RL, the distribution pattern of AVR1-CO39 in isolates from different hosts was singled out. This was consistent with the previous results that almost all the pathogenic isolates of *M. oryzae* lack *AVR1-CO39*, so they showed strong pathogenicity to *CO39* rice cultivars [62]. Second, *AVR* genes only appeared in individual strains of *M. oryzae* from different hosts, including *AVR-Pii* and *PWL1*. Among them, *PWL1* was only distributed in four *Eleusine*-infecting isolates, while all rice isolates did not contain this gene. The third distribution pattern of *AVR* genes was contrary to frequency of *AVR1-CO39*, these genes appeared more frequently in RL than in NRL, including *AVR-Pia*, *AVR-Pik*, *AVR-Pita*, *PWT4*, *PWL2,* and *AVR-Pib*. In particular, *AVR-Pia*, *AVR-Pik,* and *AVR-Pita* were absent in all wheat-, *Lolium*-, and *Bromus*-infecting isolates. The last distribution pattern included the remaining four *AVR* genes of *PWT3*, *AVR-Piz-t*, *AVR-Pi9,* and *ACE1*. It could be seen that these four *AVR* genes were widely distributed in *M. oryzae* from different hosts except *Digitaria sanguinalis* and *Pennisetum americanum*. Compared with the sequences in RL, *PWT3* containing an insertion sequence of about 394 bp in NRL was basically consistent with the previous report [63]. Similar variations of *AVR-Piz-t* were observed among rice and non-rice isolates. *AVR-Piz-t* was conserved in 35 of 62 rice strains, while SNP and InDel mutations were found in all non-rice strains. Although the relationship between the distributions and variations of different *AVR* genes in different strains seemed to be complicated, the various genotypes of *Avr1-CO39*, *AvrPik* and *AvrPita* indicated their important roles in host differentiation of *M. oryzae*.

### 3.4. Gene Pool Analysis between RL and NRL

**Figure 4:**
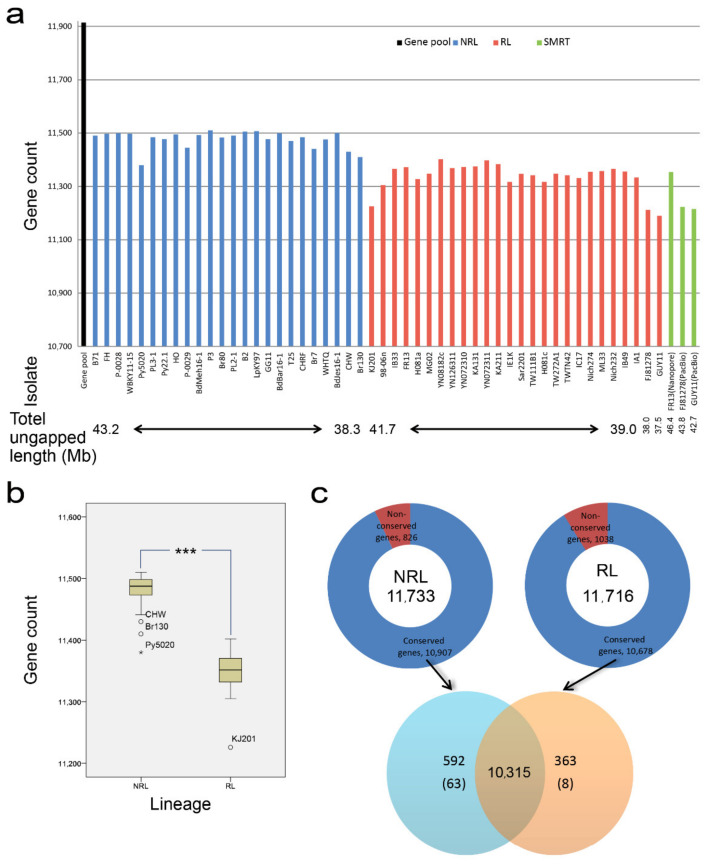
Gene pool analysis. (**a**) The distribution of the genes in gene pool shared in each isolate of RL and NRL. The black, blue, red, and green columns represent the number of genes in the gene pool, isolates of RL, isolates of NRL, and 3 rice-infecting genomes by SMRT, respectively. (**b**) Boxplot of the genes in RL and NRL. The symbol ★ indicated Py5020 in NRL with the extreme value of number of genes. However, Py5020 contained more genes than 87.5% rice-infecting isolates. The symbol ° represented the outliers CHW and Br130 in NRL, which also contained higher number of genes than all isolates of RL except YN08182c. ***, *p* < 0.001 by Student’s *t*-test. (**c**) The numbers of homologous and non-homologous genes within the group, as well as common genes shared between groups and grouping-specific genes. The number in the intersection of the two solid circles represented the common genes shared in all 48 isolates. Combination 592(63) indicated that 592 genes were common in NRL but absent in at least one isolate of RL, and 63 genes only appeared in all isolates of NRL. 363(8) indicated that 363 genes were common in RL but absent in at least one isolate of NRL, and 8 genes only appeared in all isolates of RL.

In order to show the selection pressure from various hosts on *M. oryzae* population structure at larger scale such as PAV, the gene pool was constructed with 48 *M. oryzae* genomes (Table S1). Among these genomes, wheat-infecting isolate BdBar16-1 contained the most predicted genes (11,263), while rice-infecting isolate H081c contained the least predictive genes (10,422). The average numbers of predicted genes in RL and NRL were 10,578.7 and 10,779.3. The average lengths of predicted proteins in RL and NRL were 504 and 500.3 amino acids, respectively. By BLASTP, the predicted genes from 48 genomes were merged into the whole gene pool and 11,914 genes were obtained after removal of duplicate homologous genes (Figure 4a). Then, TBLASTN was performed to obtain the common genes shared in gene pool and every genome (Figure 4b). Our results showed that the number of shared genes in every member of NRL is more than the number of genes in different isolates of RL, except that in Py5020. Moreover, the average number of genes also showed significant differences between NRL (11,477.8) and RL (11,350.2).

To clarify the effects of different sequencing and assembly methods on gene pool results, the top 24 genomes with the total ungapped length of *M. oryzae* were selected in NCBI database (Figure 4a, Table S1). It could be seen that there was no relationship between the number of genes shared in the gene pool and genome size. Additional isolates FR13, FJ81278, and GUY11 were added because of their genomic data generated by both NGS (second-generation sequencing) and SMRT (single-molecule real-time sequencing), which can be used for further comparison and evaluation. Although the genome sizes of FR13, FJ81278, and GUY11 based on NGS were less than those generated by SMRT (6.6 Mb, 5.8 Mb and 5.4 Mb, respectively), the variance in number of genes generated by these two sequencing techniques were 18, 12, and 25 in FR13, FJ81278, and GUY11, respectively, which could be neglected compared with the average number of genes (11,350.2) in RL. Furthermore, Spearman correlation analysis was applied between the number of shared genes and the four genomic features used for evaluation of the genome assembly quality (Table 2 and Table S3). When considering the whole gene pool, our results showed that the number of shared genes contained in the genome was significantly correlated with the total length and total ungapped length of genome, which is independent with the number of sequences and N50. However, total length and total ungapped length were also significantly correlated with different hosts, and similar results were obtained by Mann Whitney U test. The number of sequences and N50 between the groups of different hosts were not significantly different. Moreover, there was no relationship between the number of shared genes contained in the genome and the four parameters when RL and NRL were regarded as separate groups. It was concluded that there were significant differences in the genome size and the number of genes between RL and NRL. This may also be related to the adaptation of rice-infecting isolate to hosts.

In addition to the gene pool mentioned above, we also divided the 48 *Magnaporthe* isolates into two groups of RL and NRL. Our results showed that the number of genes in NRL (11,733) was similar to that in RL (11,716, Figure 4c). There were 10,678 conserved genes and 1038 non-conserved genes in RL, and 10,907 conserved genes and 826 non-conserved genes in NRL, respectively. While the number of conserved genes in RL is bigger than that in NRL, the number of non-conserved genes in RL is less than that in NRL. Among the gene pools containing 11,914 genes of all 48 isolates, 10,315 genes were found in the genomes of all isolates of RL and NRL, while 1599 PAV genes were absent in at least one isolate. We also compared the differences in gene pool analysis between RL and NRL with the same population size after BdJes16-1, BdMeh16-1, and BdBar16-1 removal, and similar results were obtained (Figure S10). The PAV dataset was used to screen candidate genes associated with host adaptation of *M. oryzae*.

### 3.5. Genes Associated with Host Adaptation of M. oryzae Identified by PAV Calling

**Figure 5:**
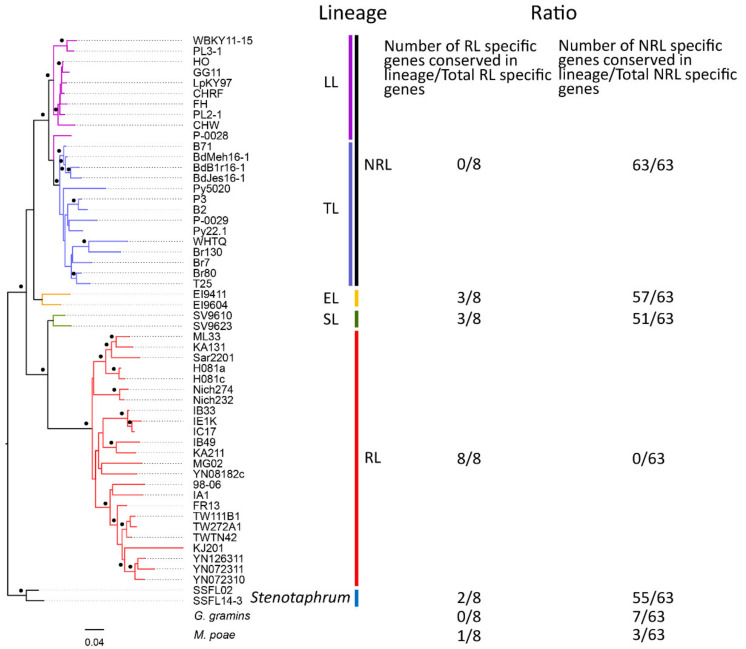
Maximum-likelihood phylogenetic tree, and occurrence ratio of RL and NRL specific genes in different lineages and two related species of Magnaporthaceae. The maximum-likelihood tree was constructed based on 1599 identified PAVs. Nodes with more than 90% bootstrap support were marked by dots. The isolates of different lineages were labeled with different colors according to the result of Figure S1. SL represented the *Setaria* lineage.

Among 1599 specific genes, there were two genetic variation patterns, one was the genes only presented in one of RL and NRL but absented in another, the other was the genes with significantly different occurrence frequencies between RL and NRL. Fortunately, we found 8 and 63 genes (with the highest sequence identity less than 50% in the other group, Table S4) completely belonging to all isolates in RL and NRL, respectively. Maximum-likelihood methods were used to construct the polygenetic trees of 48 previous isolates and 6 extra isolates based on 1599 identified PAV genes. Among these 6 isolates, 2 *Stenotaphrum*-infecting isolates were regarded as an outgroup, 2 isolates of EL and 2 isolates of *Setaria* lineages (SL) were close to rice and wheat isolates, respectively. As showed in results, except for that P-0028 was closer to isolates of TL at this time, the clusters of 54 Magnaporthe isolates based on PAV data were consistent with those based on SNP data (Figure 5 and Figure S1) and previous studies [13]. In addition, the number of 8 grouping-specific genes of RL found in other lineages of *M. oryzae* decreased with the increase of genetic distance to the rice isolates. Similarly, the number of 63 grouping-specific genes of NRL found in EL, SL, and *Stenotaphrum* lineages decreased with the increase of genetic distance to TL and LL. However, a few of 71 grouping-specific genes were found in *G. graminis var. tritici* and *M. poae*, indicating that most grouping-specific genes occurred after the differentiation of *M. oryzae*, *G. graminis var. tritici,* and *M. poae*. BLASTP and TBLASTN searches against the NCBI non-redundant database revealed that 8 grouping-specific genes of RL were orphan genes for *M. oryzae* and 11 of 63 grouping-specific genes of NRL had homologs in other species, such as *Colletotrichum sp*. According to previous reports [12,31], the expressions of 25 grouping-specific genes of NRL were induced during the compatibility interaction between wheat and wheat blast (Table S5), and 7 grouping-specific genes of RL were regulated during the development and infection in *M. oryzae* (Table S6). Therefore, our results suggested that these 71 grouping-specific genes not only completely participated in host adaptability among various lineages of *M. oryzae*, but also played an important role in pathogenicity.

**Figure 6:**
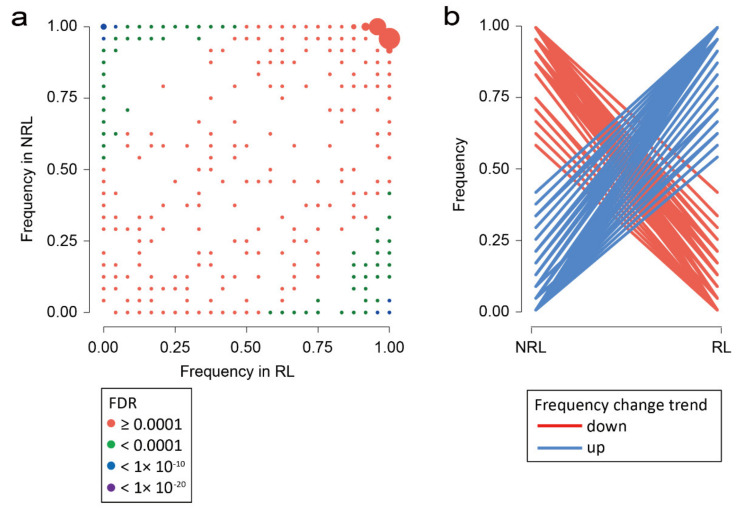
Gene selection preference by rice and non-rice hosts. (**a**) Scatter plots show occurrence frequencies of 1599 genes with PAV in RL and NRL. The different colors of the points represented the different value ranges of FDR, and the size of circle represented the number of genes with the same occurrence frequency. (**b**) Two kinds of occurrence frequency patterns of the putative selected genes by rice and non-rice host.

As for the screening of partial selection genes, the genes with higher occurrence frequencies in RL than those in NRL were regarded as favorable genes for RL and those in opposite situations were favorable genes for NRL. Our results showed that 60 and 168 favorable genes for RL and NRL were identified, respectively (Figure 6a,b, Table S4). Except for 71 complete selection genes, there were 52 and 105 favorable genes for RL and NRL respectively, indicating that these genes were undergoing the partial selection by different hosts.

To determine the putative function of 60 and 168 favorable genes for RL and NRL respectively, EggNOG database was used for GO annotation. However, there were only 14 and 31 favorable genes with GO annotation, indicating that the functions of most genes were unknown. These 45 annotation genes were classified according to previous reports (Table S7). We found that these genes were involved in various functions including recognition, cell wall modification, compound metabolism, transcript regulation, post-translational modification and so on. However, it is hard for us to enrich these genes with similar functions, the genes belonging to cell wall modification, transport, transcription factor, as well as compound metabolism might play an important role in host differentiation. Although almost all of these PAV genes do not have annotations, it could be testified by further experiments. Our analyses provided the vital information for genetic divergence and host adaption of pathogens, and these candidate genes could be the important candidates for preventing emergence of new virulence isolate in the future.

To determine the putative function of 60 and 168 favorable genes for RL and NRL, EggNOG database was used for GO annotation. However, there were only 14 and 31 favorable genes with GO annotation respectively, indicating that the functions of most genes were unknown. These 45 annotation genes were classified according to previous reports (Table S7). We found that these genes were involved in various functions including recognition, cell wall modification, compound metabolism, transcript regulation, post-translational modification, and so on. However, it is hard for us to enrich these genes with similar functions, the genes belonging to cell wall modification, transport, transcription factor, as well as compound metabolism might play an important role in host differentiation. Although almost all of these PAV genes do not have annotations, it could be testified by further experiments. Our analyses provided the vital information for genetic divergence and host adaption of pathogens, and these candidate genes could be the important candidates for preventing emergence of new virulence isolate in the future.

## 4. Discussion

### 4.1. Comparative Genomes Analyses Present the Genome Regions Involving Host Selection between RL and NRL

For crop pathogens, the domestication of hosts and the established time of co-evolution between hosts and pathogens might determine the pathogen genetic diversity. Previous studies have showed that there is no evidence that multi-speed genome evolutions are found in different fungi of the Magnaporthaceae family [64], but the nucleotide diversity (*π*) of *M. oryzae* from different hosts is various. Rice with high domestication exerts high selection pressure on *M. oryzae*, which makes rice-infecting isolates with lower genetic diversity than that from other hosts. The isolates from *Setaria* have more than twice the *π* value of the RL in both the gene coding (synonymous) and non-coding regions [51]. When using single copy genes to calculate diversity, the wheat lineage was almost two times larger than rice lineage, while the *Lolium* and *Setaria* lineages were close to rice lineage [13]. In our studies, the genetic differences of 48 *Magnaporthe* isolates from rice, wheat, *Lolium,* and *Bromus* were analyzed by comparative genomics, which were collected from 12 different countries in America, Asia, Europe, and Africa from 1988 to 2016. The diversities of isolates from non-rice hosts including *wheat*, *Lolium,* and *Eleusine* were significantly higher than that of rice-infecting isolates, which was similar to previous results. However, our results presented more detailed information on the regions involved in host differentiation combining with *F*_st_ and Tajima’s *D* calculations.

To our surprise, a large number of S-SNPs (119,637) were distributed in most windows (87.4%) which mainly led to 34.9% of windows with *F*_st_ > 0.9 and developed high degree of genetic differentiation between RL and NRL. However, such a large genetic differentiation with high value of *F*_st_ was rare in the comparison among other non-rice lineages (Figure S9a,b). Gladieux et al., pointed out that when the population divergence is older, more regions of divergence (with high *F*_st_) will appear in the genome than similar regions (with low *F*_st_) [65]. From the distribution of *F*_st_, it could be seen that the divergence of ancestors of RL and NRL was older. Tajima’s *D* is often used to distinguish the DNA sequences with random and non-random evolution [66]. When Tajima’s *D* > 0, it can be used to infer bottleneck effect or balancing selection, and on the contrary, it refers to group size enlargement or directional selection. Through comparative analysis, the windows with Tajima’s *D* < 0 in RL and NRL accounted for more than 70% and the large genetic difference between the two groups indicated that the selective sweep promoted the subdivision within a species [23,24,25,26]. While there were also significantly enriched windows with Tajima’s *D* > 0 and higher diversity in NRL, it suggested that balancing selection not only significantly increased the diversity of NRL in these regions, but also reduced the *F*_st_ between RL and NRL. Therefore, two kinds of different signatures took place between RL and NRL in whole genomes. One is that both groups were strongly affected by the selective sweep in most chromosomal regions where a large number of S-SNPs and alleles were found. Both RL and NRL were developing toward the individual direction of homogenization which led to strong genetic differentiation. The other one is that a few regions with high diversity appeared in NRL because of balancing selection, while RL retained homogenization in these regions. Similar results appeared in the population genetic statistical analysis of *Neurospora crassa* [67], and this study also showed that there are genes, which related to local photoperiod adaption of *Neurospora crassa* populations from different latitudes, in the genomic island similar to the second region with high diversity and Tajima’s *D* > 0 mentioned above. By marking the location of the favorable genes related to host adaptability of RL and NRL in the reference genome, it can be seen that most of these genes were distributed in the region with high diversity and Tajima’s *D* > 0 (Figure 1b).

Earlier studies have shown that balancing selection increases variability within a population which is an important mechanism to maintain population diversity [19,26]. However, directional selection like crop domestication reduces the diversity [28,68,69]. According to the pathotypes among different host infecting lineages, the rice-infecting isolates possess strict host specificity, while the isolates from wheat show broader hosts [70], implying balancing selection developing a lot of elasticity and variation in the wheat blast genome might be related with host range. Balance selection also provides more opportunities for wheat blast towards uncertain or risk direction. Moreover, much longer interaction history between rice and rice blast caused lower values of diversity and Tajima’s *D*, indicating that rice-infecting isolates might undergo genome-wide selective sweeps to promote the subdivision of *M. oryzae*.

### 4.2. Gene Pool Analysis Deepen the PAV Genes Promoting Host Specification of Rice Infecting Isolates

As gene pool analysis can distinguish the core genes and specific genes in the group effectively and comprehensively, integrated PAVs can better explain genetic differentiation. For example, genome evolution across 1011 *Saccharomyces cerevisiae* isolates found that genome evolution in wild isolates is mainly driven by the accumulation of SNP with very low frequencies, while the copy-number changes have a greater phenotypic effect than single nucleotide polymorphisms [71,72]. For host jump, the pathogen needs various determinants such as effectors and virulence proteins to overcome new host resistance. After successful colonization was established, a strong selection pressure was exerted on the pathogen to improve its performance and adapt to the new host environment. Present genes might promote the adaption to new host while absent genes might be necessary for previous host or recognized by new host which should be abandoned. Therefore, PAV analysis might be used to explain large variations which are responsible for the occurrence of new phenotypes.

According to the previous study [73], large host jumps might be associated with marked gene loss. For successful host jump, losses of unnecessary genes help fungi better colonize in the new host. Our analysis revealed the average number of genes shared in each rice-infecting isolate was significantly less than that in non-rice-infecting isolate, and the number of conserved genes in RL was also smaller than that in NRL. Moreover, we also found that the decreased gene number among the 24 isolates in RL was independent of the quality of sequencing data. A similar result can be found in the variation of tomato genomes selected by constant domestication. The number of genes in the wild-type tomato genome is significantly higher than that in the domesticated tomato, while the number of genes in the improved tomato is the least [68]. By comparing the frequencies of 1599 PAV genes between RL and NRL, we obtained 60 and 168 favorable genes for RL and NRL during co-evolution with hosts respectively, a quarter of which were probably secreted proteins. Among these 228 genes selected by hosts, 8 genes were present in each isolate of RL and absent in all wheat-, *Lolium*-, and *Bromus*-infecting isolates, while 63 genes were only found in each isolate from wheat, *Lolium,* and *Bromus*. It is important to note that there were not more than 3 (37.5%) grouping-specific genes of RL conserved in other lineages of *M. oryzae*, such as the *Eleusine*, *Stenotaphrum,* and *Setaria* lineages which possessed no less than 51 (81.0%) grouping-specific genes of NRL. In order to better adapt to the host and gain advantages in the arms race with the host, the genome of RL had undergone considerable simplification and specialization under stronger pressure, such as the loss of useless or unnecessary genes, while the selection of beneficial or specific genes was prudent and effective. Therefore, gene pool analysis suggested that, compared with the NRL, the RL experienced a higher level of natural selection through the gain or loss of gene.

### 4.3. Functions of the Genes Related to Host Adaptation

Gene pool analysis revealed 60 and 168 favorable genes for host adaption of RL and NRL respectively, but there were only 14 and 31 favorable genes with GO annotation, which indicated that the functions of a large number of genes need to be further identified. According to the annotated results and functional classification, some favorable genes for RL are likely concentrated on transport, while many favorable genes for NRL are enriched in cell wall modification, transcription factor, and compound metabolism. The studies related to the transport-related genes in *M. oryzae* which involve fungal development, xenobiotic degradation, and virulence have been reported. However, the functions of outer membrane auto transporter and importin are unknown in *M. oryzae*. According to previous results, importin family proteins, importin α and β play different roles in cargoes with nuclear localization signal transport from cytoplasm to nucleus. Importin α acts as an adaptor to bind importin b and cargo to enter into the nucleus. For hosts, different importin α isoforms govern the host adaptation of influenza virus [74]. Some importin-related genes involved in oxidative stress response, pathogenicity and transport of cargoes were identified [75]. For example, the β-importin KAP8 (Pse1/Kap121) is required for nuclear import of the cellulose transcriptional regulator XYR1, asexual sporulation, and stress resistance in *Trichoderma reese*i [76].

Compared with the situation of RL, more favorable genes for NRL were obtained and annotated. Gene families related to Glycosyl hydrolase (GH) have participated in cell wall modification [77]. Chitin oligosaccharides, as important constitutions of fungal cell wall, are the representative general elicitors to induce defense responses in rice. Recognition and modification for chitin become a co-evolutionary strategy between rice and *M.oryzae*. There are many chitin-related receptors such as CEBiP and OsCERK1 with LysM motifs to induce defense reaction in rice [78], while the effector Slp1 (secreted LysM protein 1) competes with CEBiP for the binding of chitin oligosaccharides [79]. Chitinase used for chitin degradation, which are regarded as members of GH family, can be recognized by rice [80,81], while a series of proteases involving chitinase cleavage also improves the fungal virulence [82]. Therefore, the modification of chitin and chitin-related enzymes is usually regarded as a strategy to escape recognition of rice. Moreover, the favorable gene encoding pectinesterase in our results is also involved in host specialization of *Epichloë* [83], indicating that the genes related to cell wall play a universal role in host differentiation among fungi. We also found a favorable gene for NRL encoding conserved glycine-rich protein (GRP), and previous report identified GRP1 as a splicing factor to regulate post-transcription and fungal virulence and growth in *M. oryzae* [84]. Furthermore, two avirulence effectors *PWL1* and *PWL2* containing glycine-rich and hydrophilic domains prevent the rice-infecting isolates from infecting weeping lovegrass with host specificity, indicating that conserved glycine-rich proteins might be recognized by the host [53,54].

Among the favorable genes for NRL, two of which encoding heterokaryon incompatibility protein (HET) were screened. Heterokaryon incompatibility in filamentous fungi is regarded as a rejection response for genetically different entities. Heterokaryon incompatibility is controlled by het genes, whose incompatible alleles co-expressed in a common cytoplasm initiate cell death [85]. Coincidentally, two genes containing caspase structural domain were obtained in the favorable genes for NRL, and the induction of caspase can be used as the biomarker of programmed cell death [86]. Compared with favorable genes of RL, these genes mentioned above might be associated with the adaption to host species.

### 4.4. The Gain or Loss of AVR and Effector Affects the Host Differentiation of M. oryzae

Many genes of *M. oryzae* are under positive selection and most of them are unique sequences compared with other *Sordariomycetes*. Moreover, in order to gain an advantage in the interaction between host and pathogen, *M. oryzae* contains more secretory proteins and effectors than other *Sordariomycetes* [87]. *AVR* genes have been reported to involve in recognition for different hosts by *M. oryzae*. For instance, polymorphisms of *Pik* and *AVR-Pik* alleles suggest that the mutant of *AVR* genes drives the fast adaption of *M. oryzae* to new resistant varieties [88]. In addition, *AVR-Pia* has been identified as a possible determinant of the specialization of *M. oryzae* to local japonica rice in rice production area of Yuanyang county [89].

*PWL2* identified from weeping lovegrass participates in host jump of *M. oryzae* among different crophosts [54]. In this study, our results showed that the presence or absence variations of *AVR1-CO39*, *AVR-Pita,* and *AVR-Pik* involved host differentiation, especially between rice blast and wheat blast. However, the relationship between *AVR* genes and host adaption seems not strict due to different genomes used for analysis [90]. As expected, the effectors showed the richest diversity in all the genes of *M. oryzae* [51] and many PAV genes with signal peptide were identified from our analysis, which indicated that there might be potential effectors responsible for host recognition.

For hosts, the cognate *R* genes are considered as the effective defense weapon to pathogens. Almost all of identified *R* genes encoding NBS–LRR proteins seem conserved in animals and plants [91]. Moreover, some *R* genes from maize, sorghum, and brachypodium confer the resistance to rice blast, indicating that the abundant rice blast *R* genes occur not only within species but also among species [92]. On the one hand, the host jump means the pathogens possess enough resource to overcome the resistance from new hosts. On the other hand, the hosts might not be ready or have no ability to cope with novel pathogens. Domestication of crops toward preference traits often loses some genetic resources which increase the risk to face unknown challenges for hosts. When the wheat blast disease has not been reported, it is difficult to determine whether the R genes against *M. oryzae* might be lost in wheat due to neglect of potential value and domestication of wheat. Therefore, the loss of genetic diversity in hosts should be considered as an alert for host jump.

## Figures and Tables

**Table 1:** Diversity in intronic regions and intergenic regions among different linages.

**Lineage**	**Intronic Regions**	**Intergenic Regions**
RL (*n* = 24)	0.0002665	0.0005304
NRL (*n* = 24)	0.0021783	0.0035670
TL (*n* = 14)	0.0021997	0.0033026
LL (*n* = 10)	0.0016510	0.0029569
EL (*n* = 7)	0.0032822	0.0050494

**Table 2:** Correlation analyses between four genomic features and the number of shared genes in each isolate and hosts.

**Isolates**	**Features**	**Number of Genes**		**Hosts (Rice or Non-Rice)**				
		**Spearman ^a^**		**Spearman ^b^**		**Mann-Whitney U Test ^c^**		
		**ρ**	**Sig**	**ρ**	**Sig**	**U**	**Z**	**Sig**
48 isolates of RL and NRL	Total length	0.626 **	0.000	0.767 **	0.000	33.000	−5.258	0 **
	Total ungapped length	0.693 **	0.000	0.752 **	0.000	38.000	−5.155	0 **
	Scaffold (contig) number	−0.079	0.595	−0.244	0.095	207.000	−1.670	0.095
	N50	−0.081	0.585	−0.066	0.655	266.000	−0.454	0.650

^a^ The Spearman correlation analysis between four genomic features and the number of genes shared in isolates. ^b^ The Spearman correlation analysis between four genomic features and hosts of *M. oryzae* (rice or non-rice). ^c^ The Mann–Whitney U tests of four genomic features between isolates of RL and NRL. **, significant correlation at the 0.01 level in Spearman correlation analysis and *p* < 0.01 by Mann–Whitney U *t*-test, respectively.

## Data Availability

The data presented in this study are available in this article and its Supplementary Materials.

## Supplementary Materials

The following are available online at https://www.mdpi.com/article/10.3390/jof8010005/s1. Figure S1: Phylogenetic tree of 48 RL, NRL isolates, 2 EL isolates and 2 SL isolates based on genome-wide SNP data, Figure S2: Statistical distribution of π and Tajima’s *D* of 4045 Calculation window in RL and NRL, Figure S3: *F*_st_, π and Tajima’s *D* for 21 isolates of RL and 21 isolates of NRL (without BdJes16-1, BdMeh16-1 and BdBar16-1), Figure S4: Statistical distribution of π and Tajima’s *D* of 4045 calculation window in RL(21) and NRL(21), and the 21 RL isolates used in this section, Figure S5: *F*_st_, π and Tajima’s *D* for RL(14) and TL, respectively, Figure S6: Statistical distribution of π and Tajima’s *D* of 4045 calculation window in RL(14) and TL, and the 14 RL isolates used in this section, Figure S7: *F*_st_, π and Tajima’s *D* for RL(10) and LL, respectively, Figure S8: Statistical distribution of π and Tajima’s *D* of 4045 calculation window in RL(10) and LL, and the 10 RL isolates used in this section, Figure S9: Statistics of *F*_st_ and π in comparison between different lineages, Figure S10: Gene pool analysis of 21 RL isolates and NRL without BdJes16-1, BdMeh16-1 and BdBar16-1, Table S1: *M. oryzae* isolates used in this study, Table S2: Statistics of SNPs number scanned by comparing 48 isolates with different reference genomes, Table S3: Correlation analyses between four genomic features and the number of shared genes in RL and NRL, Table S4: Genes detected in the 48 isolates with PAV calling and significantly different frequencies between RL and NRL, Table S5: Expression of 25 NRL Specific genes in field samples collected during wheat blast epidemic in Bangladesh 2016, Table S6: Expression of 7 RL Specific genes during the compatible interaction between 98-06 and rice plants (cv. CO-39), Table S7: Classification of genes related to host adaptation for *M. oryzae* with annotation.
