# Supplementary figures and images for: Comparative Genomics and Gene Pool Analysis Reveal the Decrease of Genome Diversity and Gene Number in Rice Blast Fungi by Stable Adaption with Rice
Source: J Fungi (Basel). 2021 Dec 22;8(1):5. doi: 10.3390/jof8010005 (PMC8778285; doi:10.3390/jof8010005)

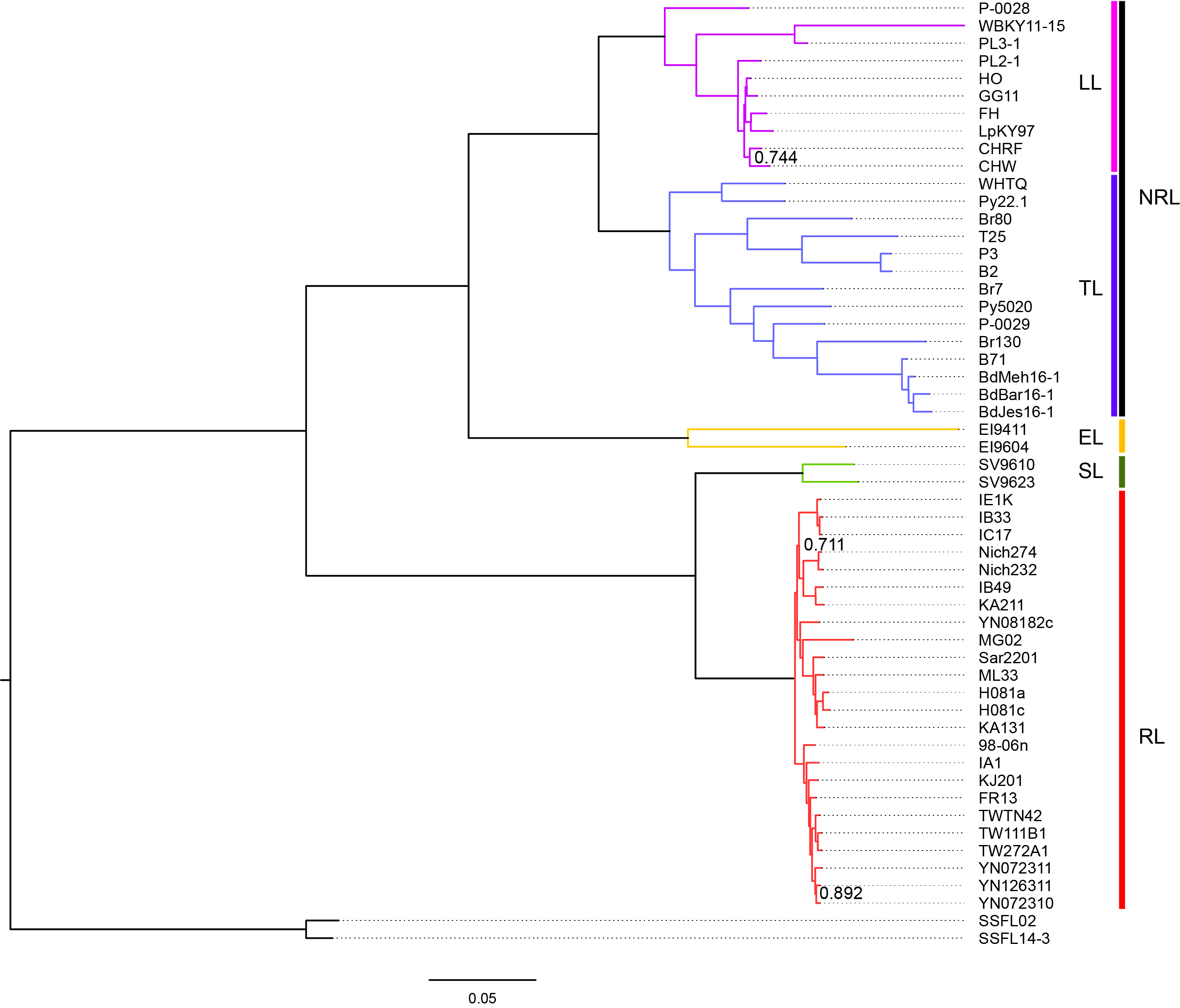

Supplement: Supplementary file 1 [file jof-08-00005-s001.zip › jof-1470822-supplementary/Figure S1.tif]

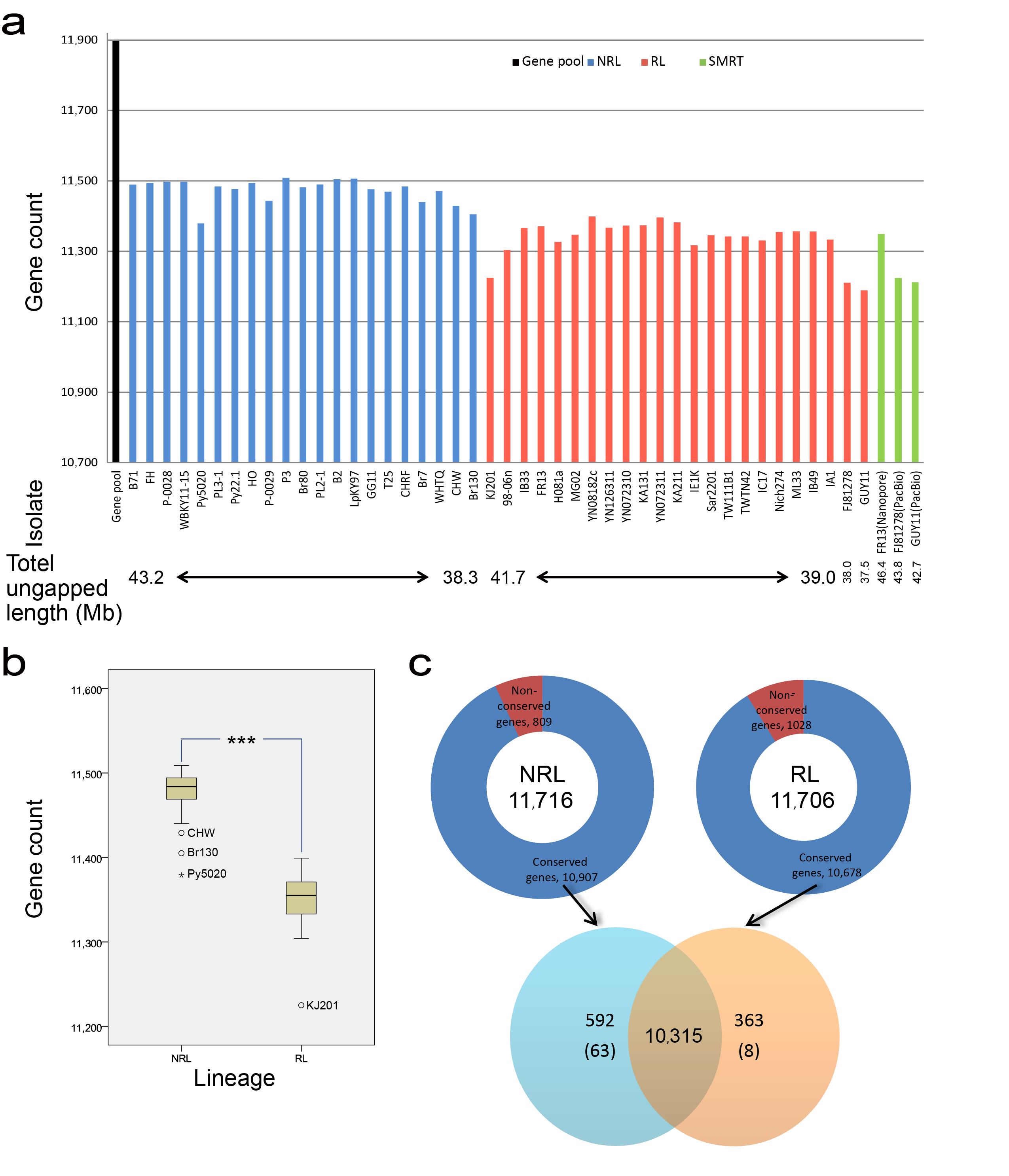

Supplement: Supplementary file 1 [file jof-08-00005-s001.zip › jof-1470822-supplementary/Figure S10.tif]

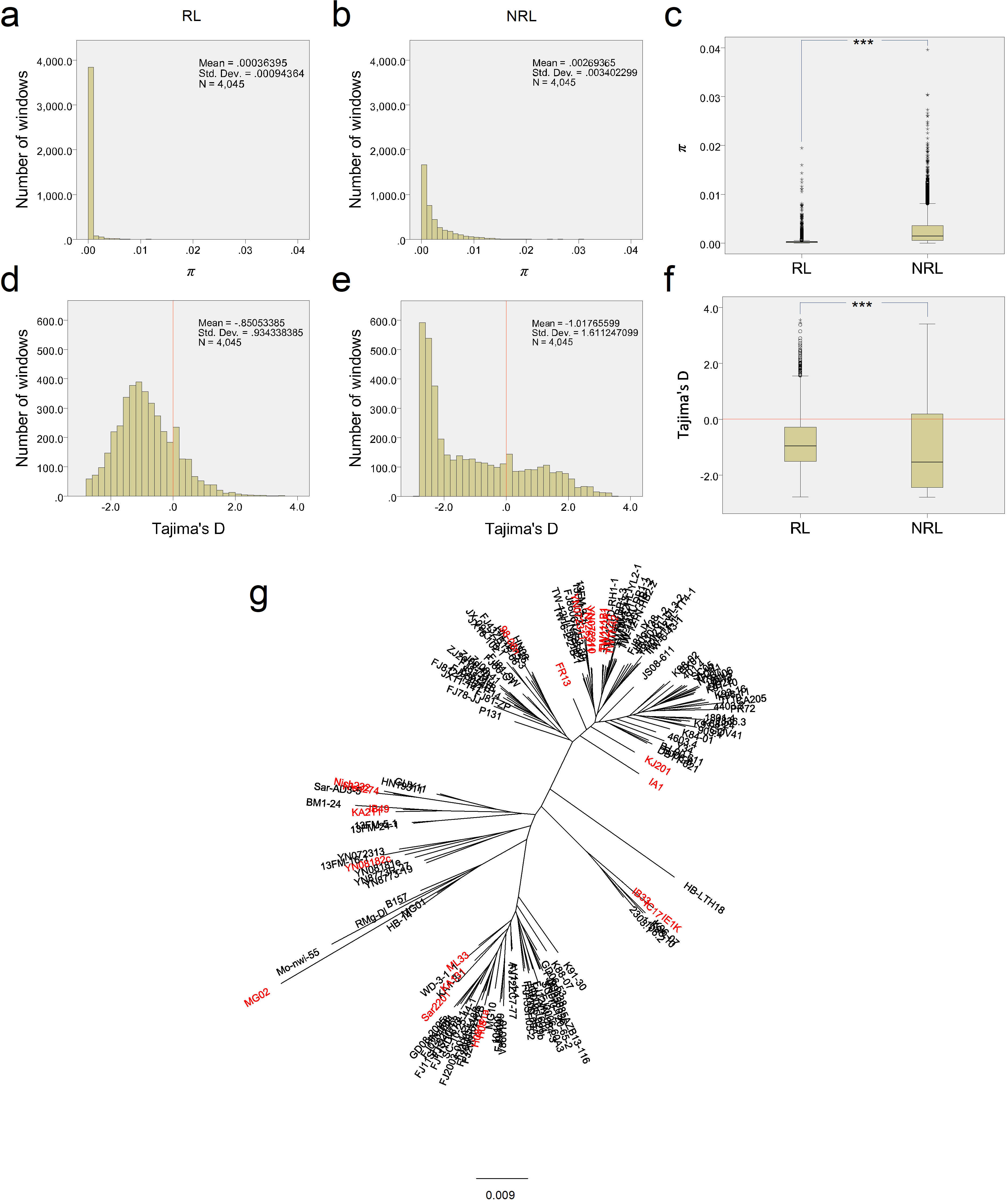

Supplement: Supplementary file 1 [file jof-08-00005-s001.zip › jof-1470822-supplementary/Figure S2.tif]

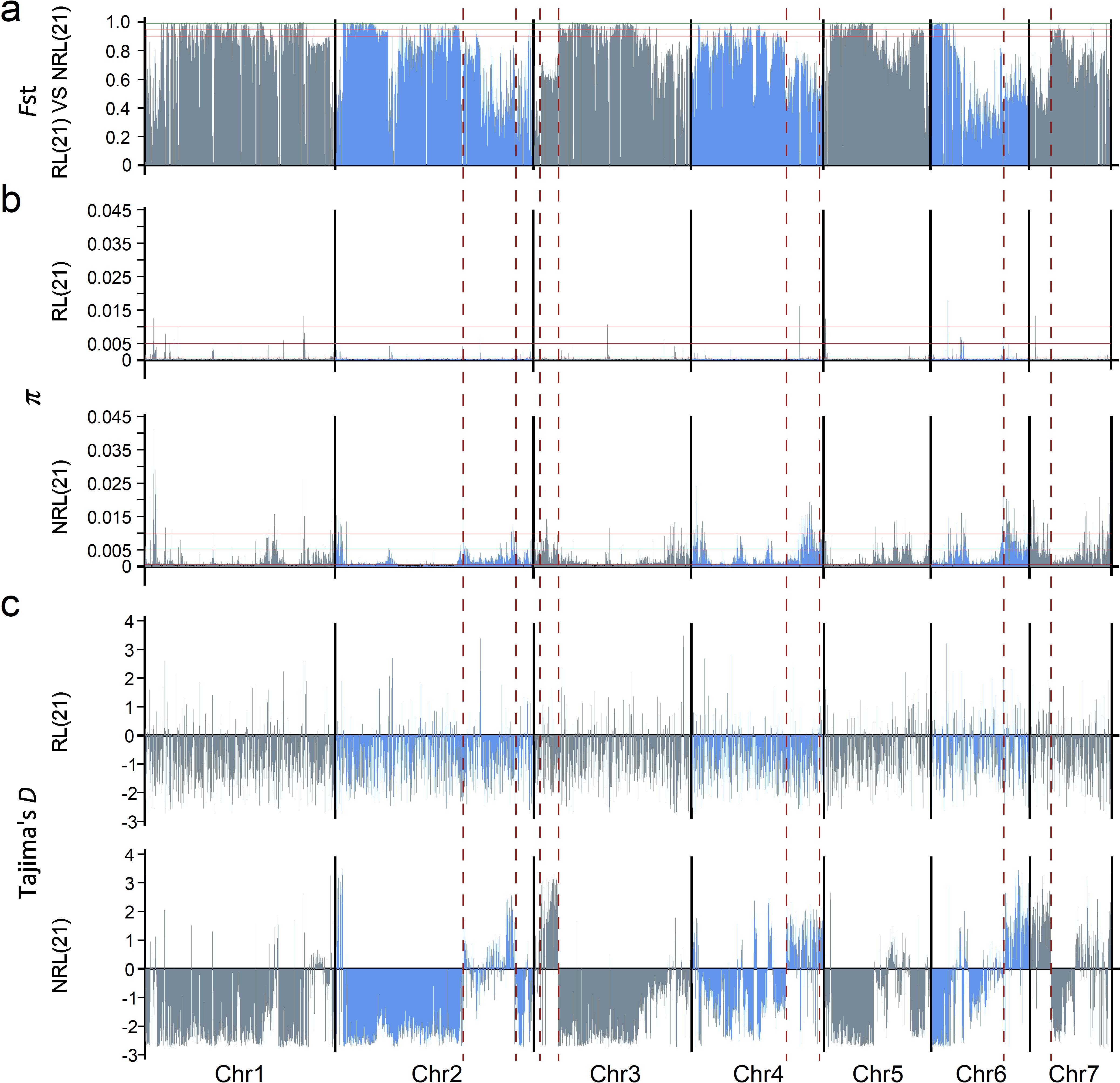

Supplement: Supplementary file 1 [file jof-08-00005-s001.zip › jof-1470822-supplementary/Figure S3.tif]

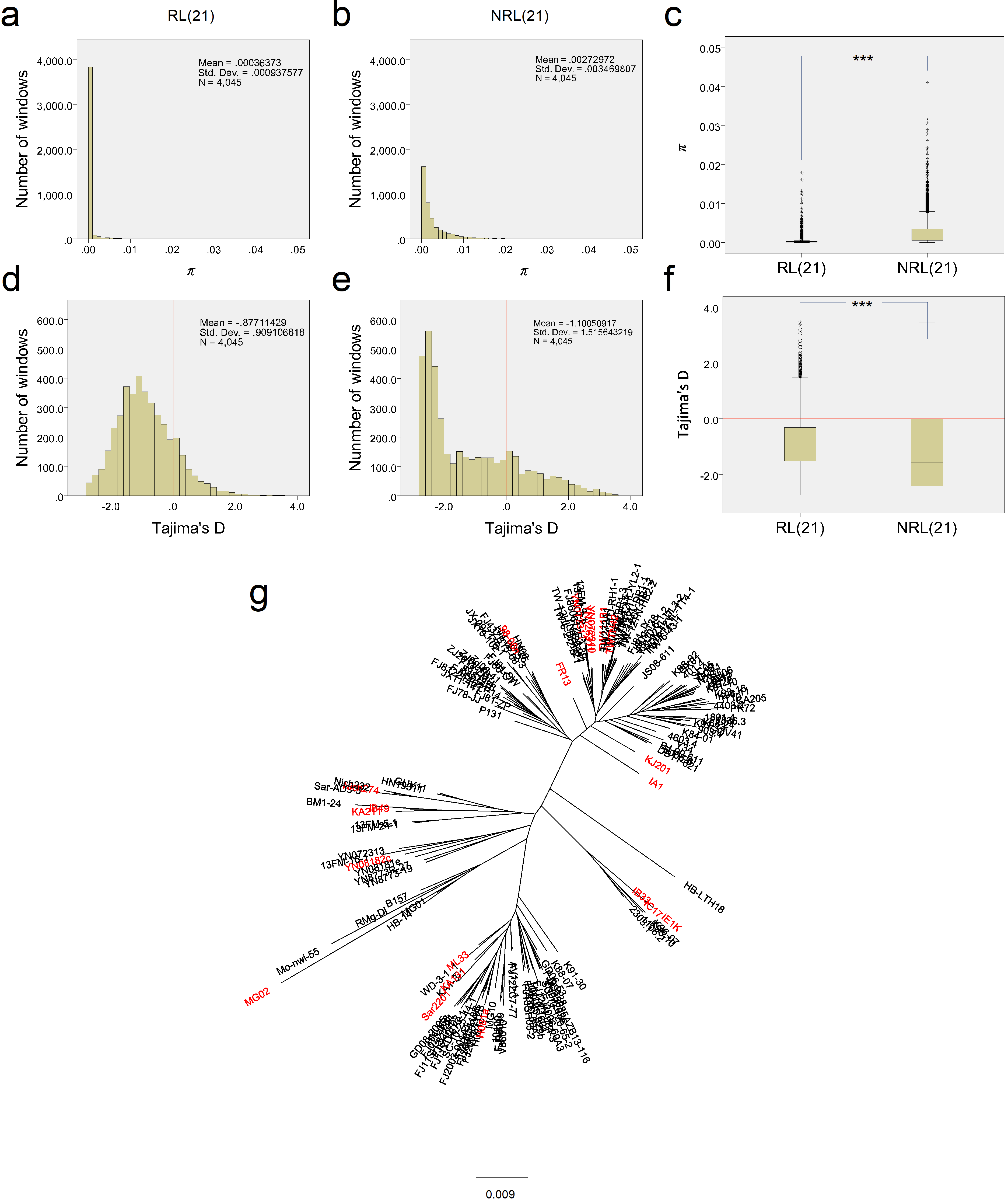

Supplement: Supplementary file 1 [file jof-08-00005-s001.zip › jof-1470822-supplementary/Figure S4.tif]

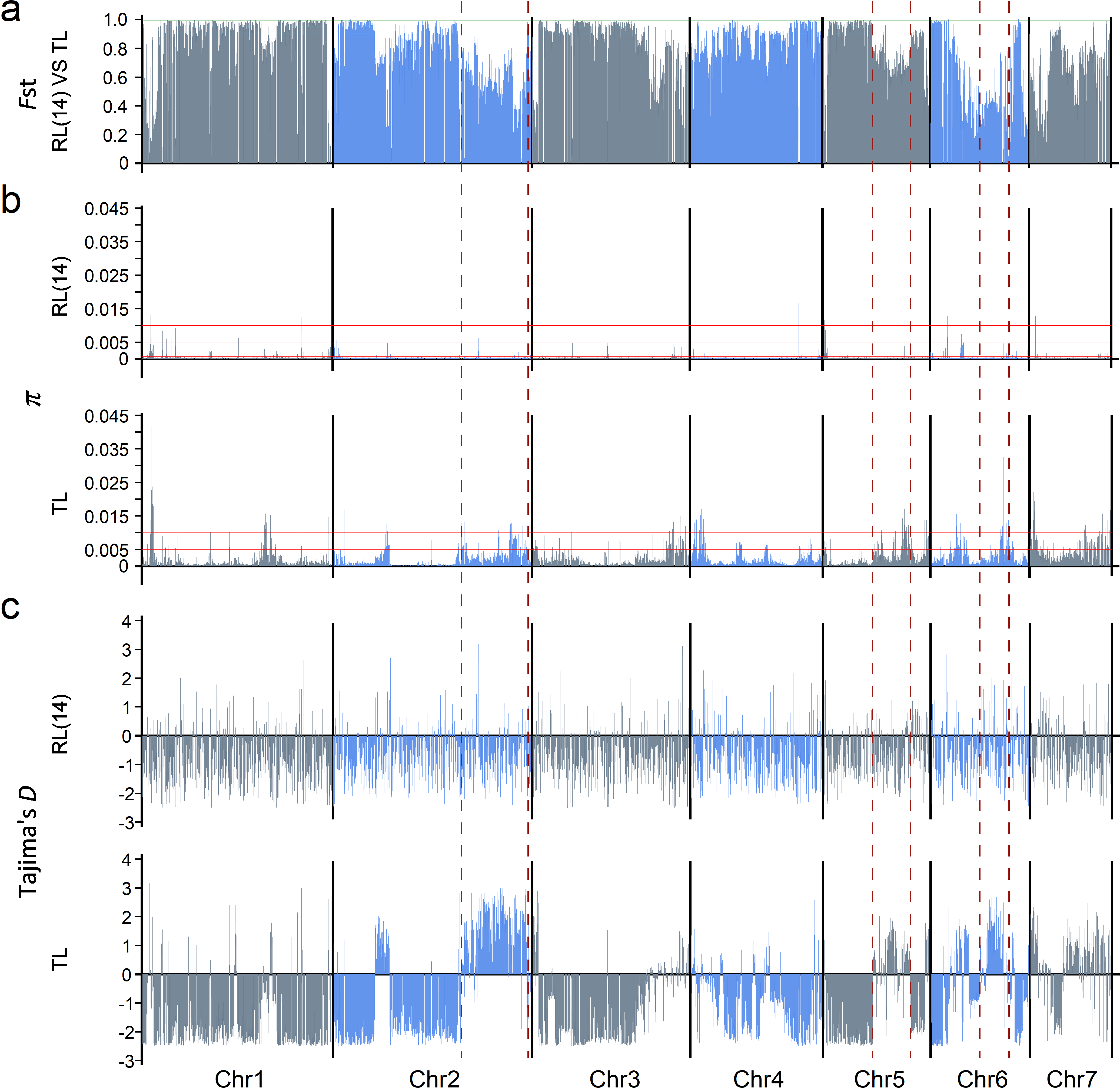

Supplement: Supplementary file 1 [file jof-08-00005-s001.zip › jof-1470822-supplementary/Figure S5.tif]

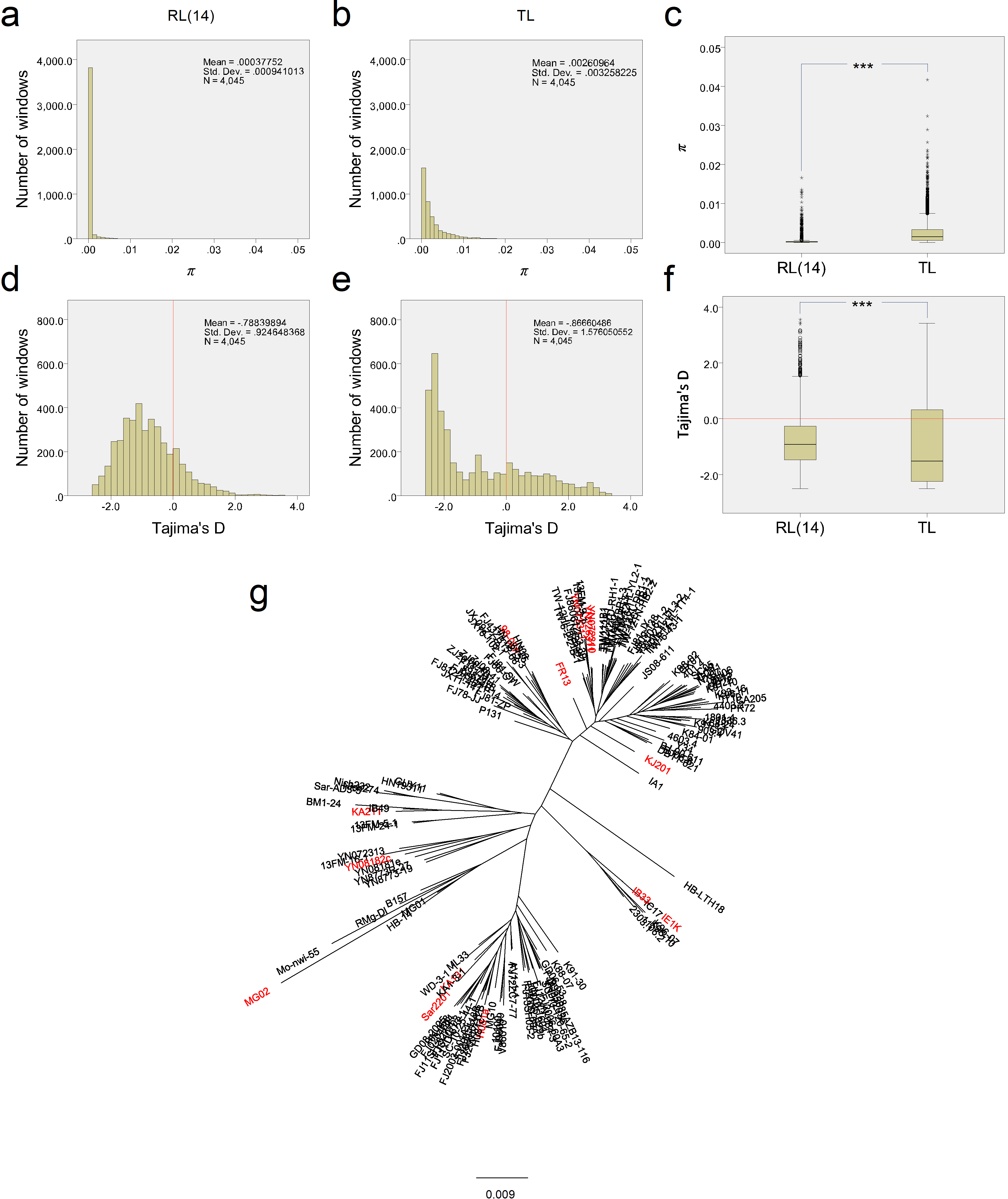

Supplement: Supplementary file 1 [file jof-08-00005-s001.zip › jof-1470822-supplementary/Figure S6.tif]

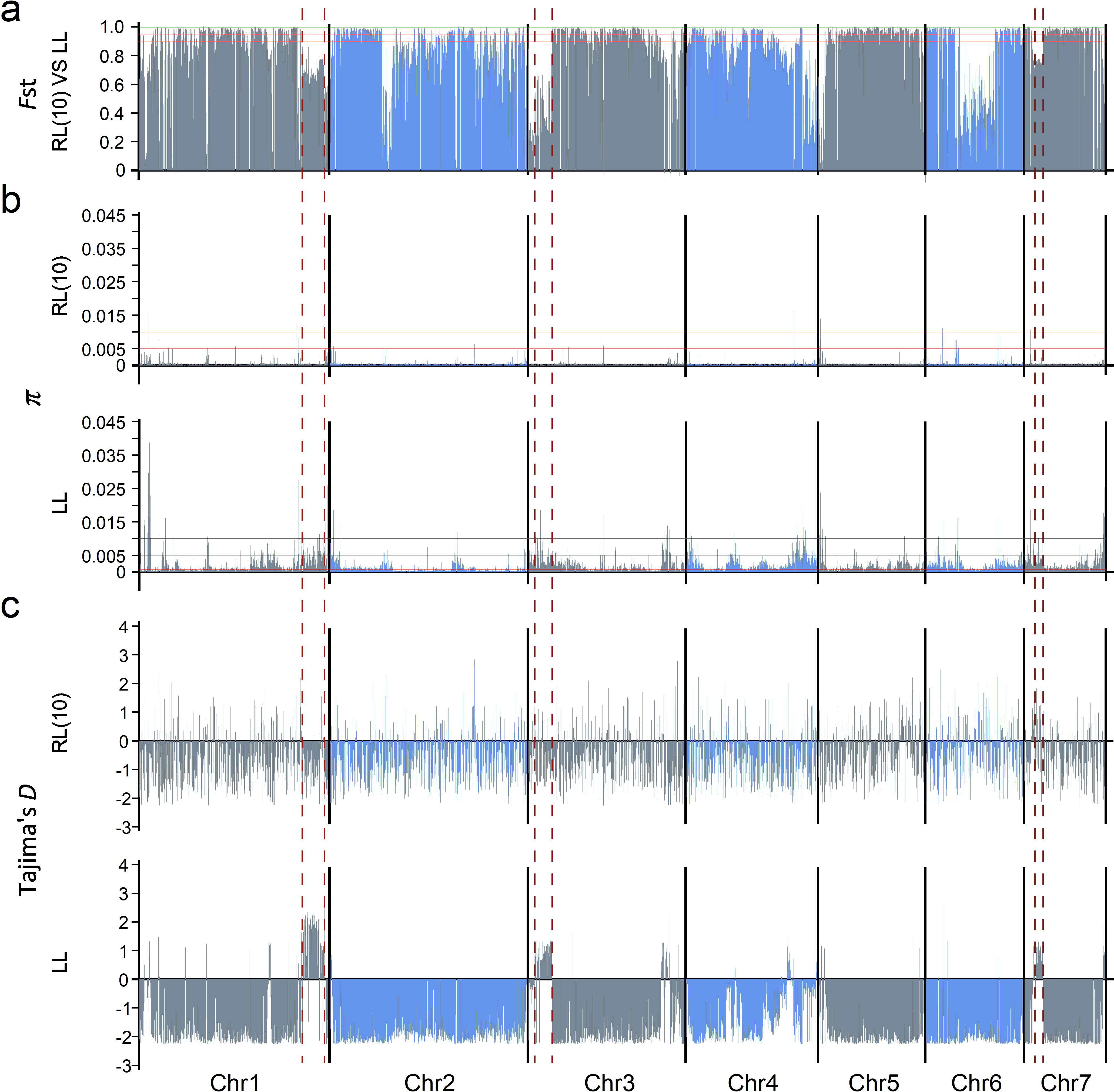

Supplement: Supplementary file 1 [file jof-08-00005-s001.zip › jof-1470822-supplementary/Figure S7.tif]

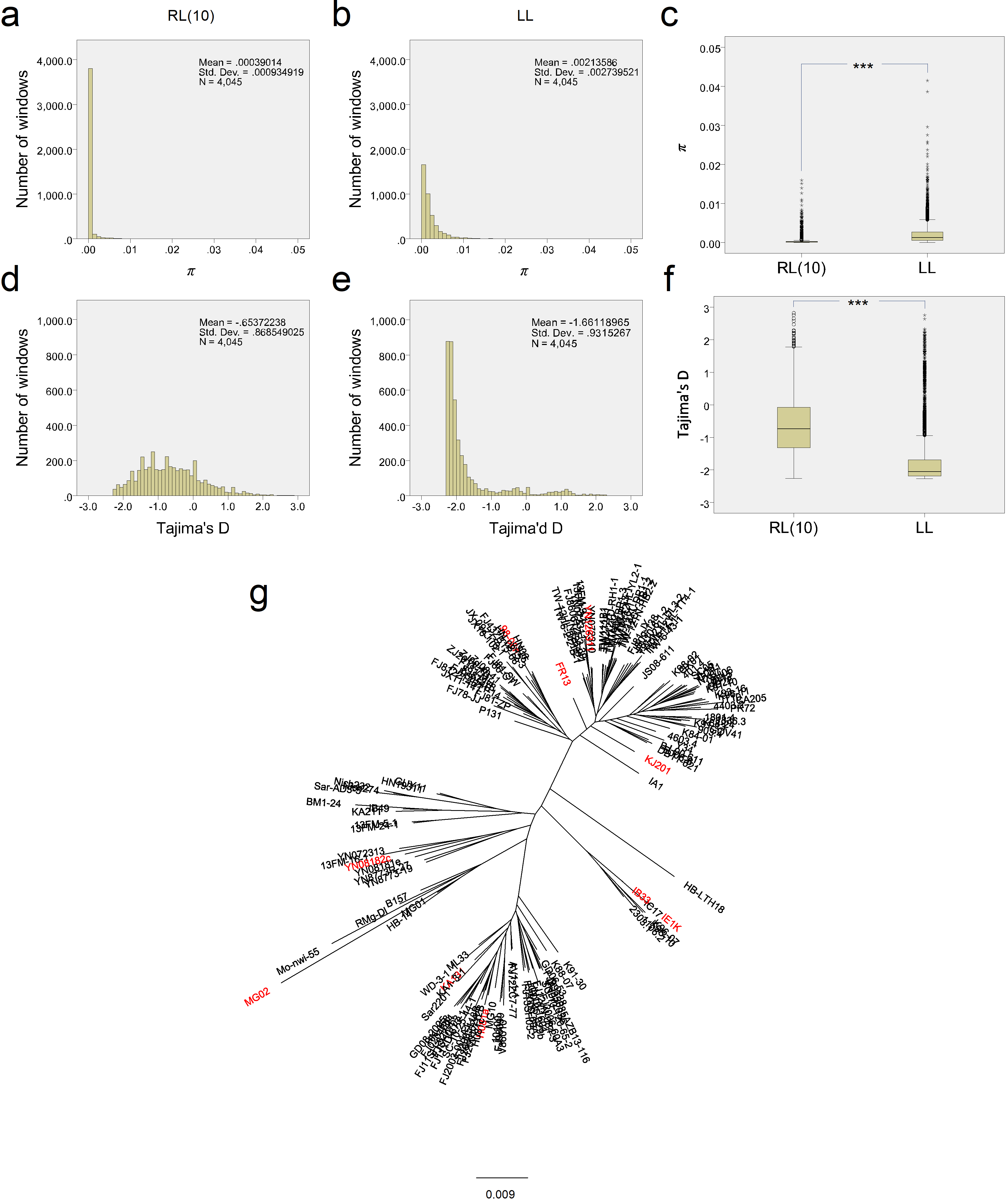

Supplement: Supplementary file 1 [file jof-08-00005-s001.zip › jof-1470822-supplementary/Figure S8.tif]

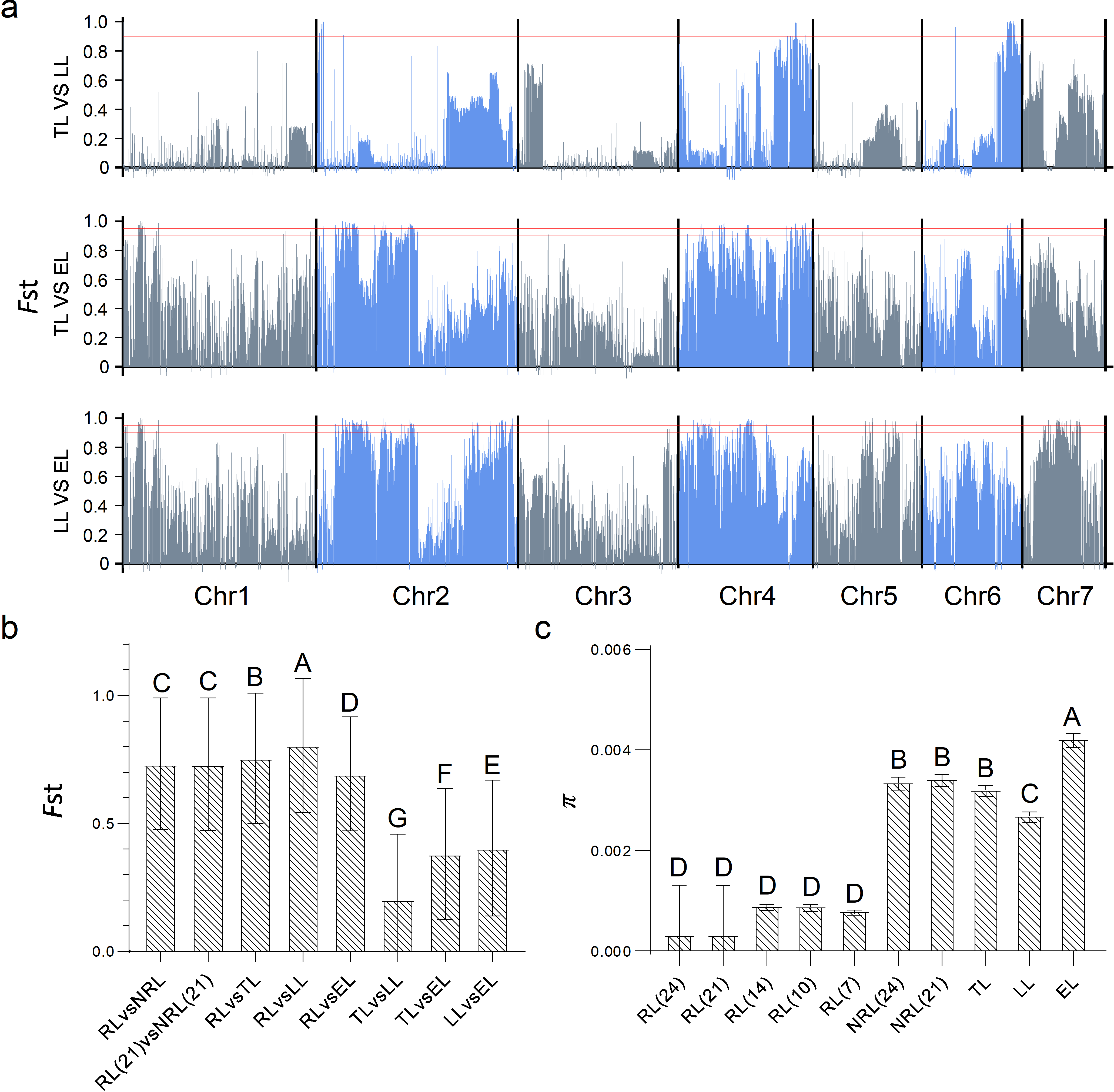

Supplement: Supplementary file 1 [file jof-08-00005-s001.zip › jof-1470822-supplementary/Figure S9.tif]
